# CULTURALLY CONCORDANT CARE: CLINICIAN PERSPECTIVES ON PROVIDING CANCER CARE TO SOUTH ASIANS

**DOI:** 10.1093/geroni/igac059.2357

**Published:** 2022-12-20

**Authors:** Nainwant Singh, Veena Manja, Ambri Pukhraj, Rashmi Risbud, Shreya Desai, Lidia Schapira, Karl Lorenz, Ranak Trivedi

**Affiliations:** VA Palo Alto, Palo Alto, California, United States; VA Palo Alto, Palo Alto, California, United States; Stanford University, Stanford, California, United States; University of California, Davis, Davis, California, United States; Veterans Affairs Palo Alto Health Care System, Menlo Park, California, United States; Stanford University, Stanford, California, United States; Veteran Affairs Palo Alto Health Care System, Stanford, California, United States; Stanford University, Palo Alto, California, United States

## Abstract

In many South Asian (SA) cultures, cancer is stigmatized, and family members are expected to become primary caregivers. Clinicians need to be familiar with these SA needs and values to provide culturally concordant care. The South Asian Family Approaches to Disease (SAFAD) study aims to understand cultural needs of SAs managing breast cancer. We conducted semi-structured qualitative interviews with multidisciplinary clinicians at a major academic medical center about caregiver interactions, cultural dynamics affecting clinical practice, unmet patient needs, and perceptions of culturally concordant care. Participants included physicians(8), a nurse practitioner(1), a social worker(1), and a physician assistant(1) with experience in palliative care(5), hematology/oncology(4), breast cancer(3), critical care(2), and radiation oncology(1) with one to 42 years in practice. Participants identified as Caucasian/White(6), South Asian(3), African American/Black(1), and Chinese(1). Clinicians noted the following: 1)SAs have greater family involvement in care and may defer treatment decisions to family members; 2)SAs seek clinician support with cancer management and nutrition; 3)SA emotions and hesitation around sensitive topics may result in non-disclosure; 4)SAs have diverse caregiver roles; and 5)individual SA needs cannot be generalized within the diaspora. “There’s still some stigma… with breast cancer… [it] may lead a patient to not want to share their diagnosis… then set up a patient for having less support… [We can] help them feel more comfortable opening up selectively.” Understanding such cultural needs is essential to cultivating trust and providing person-centered care. Interventions and resources to promote culturally concordant cancer care can target education in these areas.

